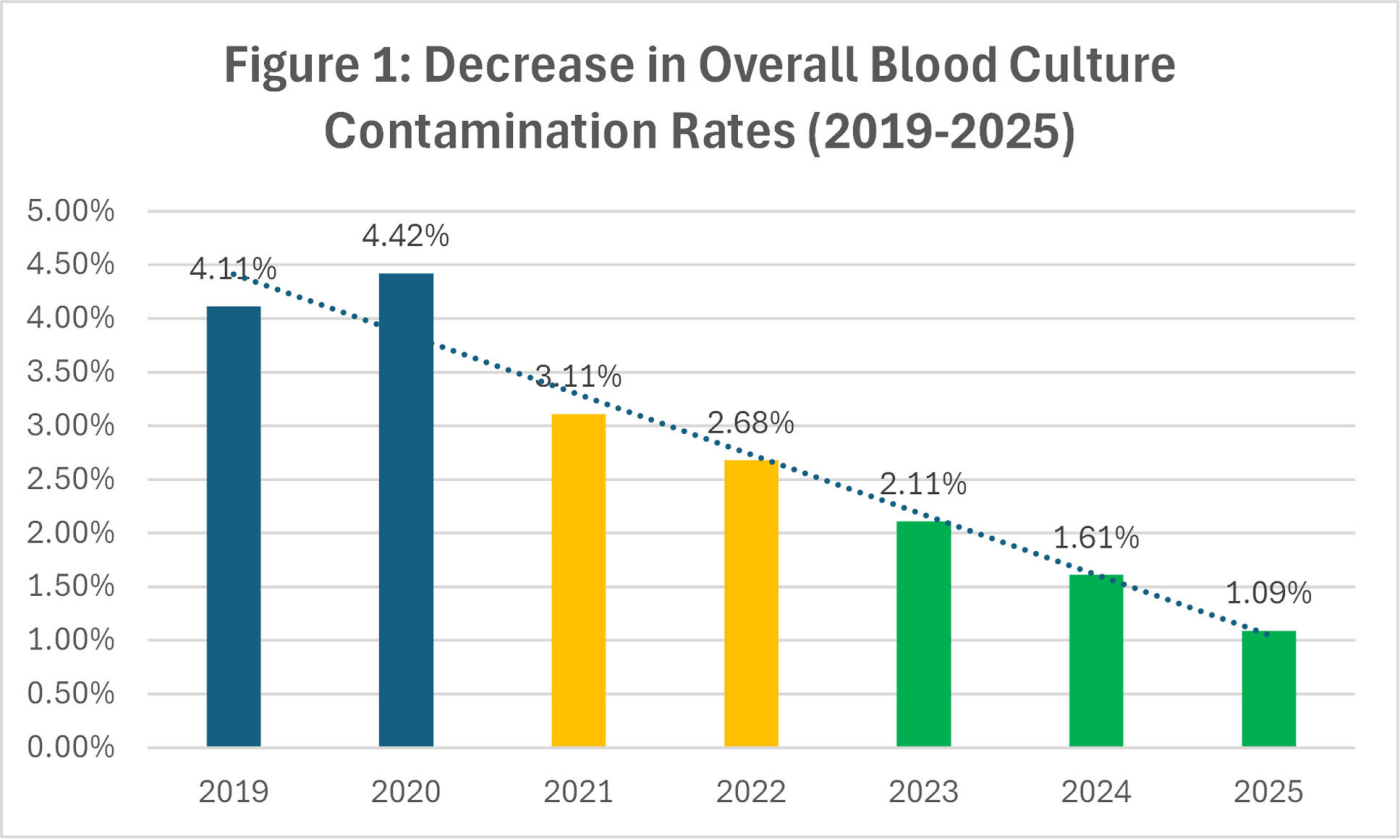# 266 Mycobacterium abscessus associated with facility-wide water contamination: An outbreak investigation

**DOI:** 10.1017/ash.2026.10734

**Published:** 2026-06-23

**Authors:** Heidi Gilbert, Karen Giuliano

**Affiliations:** 1 Stillwater Medical Center; 2 U Mass Amherst

## Abstract

**Background:** Blood culture contamination (BCC) is a persistent problem in the hospital, contributing to unnecessary antibiotic exposure, excessive diagnostic testing, increased length of stay, and higher costs. For patients with suspected or confirmed sepsis, BCC has particularly serious implications, as early identification of the causative pathogen and prompt initiation of appropriate antimicrobial therapy are critical. National benchmarks define <3% contamination as acceptable, yet emerging evidence supports a more ambitious goal of ?1%, particularly in high-risk populations such as patients with sepsis. Methods Using a sequential practice change across three time periods: (1) no blood culture diversion device (2019–2020), (2) implementation of manual blood diversion device (BDD) #1 (2021–2022), and (3) automatic BDD#2 (2023–present), we have achieved a 74% reduction in BCC (Figure 1). In this project, we further evaluated the impact of this overall reduction on patients admitted with suspected or confirmed sepsis. Results Data were extracted for adult patients admitted to the hospital who had a final discharge diagnosis of sepsis, regardless of their initial admission diagnosis. A total of 154 blood cultures were analyzed across the three time periods. During period 1 (2019 & 2020), 6/62 cultures were contaminated (9.7%). After implementation of manual BDD#1 (11/17/2021-2022), BCC decreased to 2/30 cultures (6.7%). After transition to automatic BDD#2 (1/20/2023-present), BCC further declined to 3/62 cultures (4.8%). One-way analysis of variance (ANOVA) demonstrated a statistically significant difference in BCC rates across the time periods, with post hoc comparisons showing a significantly lower contamination rate when using BDD#2 as compared with both no BDD and manual BDD#1. Additionally, manual BDD#1 was used in 48% (30/62) of the blood culture collections versus to 78% for automatic BDD#2, with staff feedback indicating greater ease of use with the automatic BDD#2 system. Conclusion Adoption of BDD was associated with a 50% (9.7-4.8%) reduction in BCC, with the lowest rates following implementation of automatic BDD#2. Compliance with BDD use was also highest with automatic BDD#2, and staff reported greater ease of use. These findings underscore the importance of optimizing BCC practices as a key component of sepsis management, antimicrobial stewardship, and patient safety efforts. Additional research is needed to identify strategies to further reduce BCC in sepsis patients, with particular attention to BDD usability, workflow integration, and process adherence during real-world use.

**Figure:**